# Bacterial Magnetosome Biomineralization - A Novel Platform to Study Molecular Mechanisms of Human CDF-Related Type-II Diabetes

**DOI:** 10.1371/journal.pone.0097154

**Published:** 2014-05-12

**Authors:** Natalie Zeytuni, René Uebe, Michal Maes, Geula Davidov, Michal Baram, Oliver Raschdorf, Assaf Friedler, Yifat Miller, Dirk Schüler, Raz Zarivach

**Affiliations:** 1 Department of Life Sciences, Ben Gurion University of the Negev, Beer-Sheva, Israel; 2 The National Institute for Biotechnology in the Negev, Ben Gurion University of the Negev, Beer-Sheva, Israel; 3 Department of Chemistry, Ben Gurion University of the Negev, Beer-Sheva, Israel; 4 Ilse Katz Institute for Nanoscale Science and Technology, Ben-Gurion University of the Negev, Beer-Sheva, Israel; 5 Institute of Chemistry, The Hebrew University of Jerusalem, Givat Ram, Jerusalem, Israel; 6 Ludwig Maximillian University of Munich, Dept. Biology I, Martinsried, Germany; Simon Fraser University, Canada

## Abstract

Cation diffusion facilitators (CDF) are part of a highly conserved protein family that maintains cellular divalent cation homeostasis in all organisms. CDFs were found to be involved in numerous human health conditions, such as Type-II diabetes and neurodegenerative diseases. In this work, we established the magnetite biomineralizing alphaproteobacterium *Magnetospirillum gryphiswaldense* as an effective model system to study CDF-related Type-II diabetes. Here, we introduced two ZnT-8 Type-II diabetes-related mutations into the *M. gryphiswaldense* MamM protein, a magnetosome-associated CDF transporter essential for magnetite biomineralization within magnetosome vesicles. The mutations' effects on magnetite biomineralization and iron transport within magnetosome vesicles were tested *in vivo*. Additionally, by combining several *in vitro* and *in silico* methodologies we provide new mechanistic insights for ZnT-8 polymorphism at position 325, located at a crucial dimerization site important for CDF regulation and activation. Overall, by following differentiated, easily measurable, magnetism-related phenotypes we can utilize magnetotactic bacteria for future research of CDF-related human diseases.

## Introduction

Metal cations are essential cellular elements [1]. However, their excess accumulation is highly cytotoxic. Cellular metal cation homeostasis is tightly regulated by diverse sensory and export systems [2]. One of these systems is the ubiquitous Cation Diffusion Facilitator (CDF) protein family [3]. CDFs typically exploit the proton motive force to transport cytoplasmic divalent metal cations such as Zn^2+^, Co^2+^, Cd^2+^, Fe^2+^, Mn^2+^ and Ni^2+^ [4]–[8]. The broad substrate spectrum of CDF transporters allows their participation in numerous biological pathways. As such, CDFs are localized to different cellular compartments, such as the Golgi apparatus of animal cells, the vacuolar membranes of yeast and plants or the bacterial membrane [9]. The association of altered regulation or mutations within several human CDFs (ZnT or *SLC30A* 1-10) with severe human diseases demonstrates the crucial role of CDFs for cellular metal homeostasis. Recent studies have described a link between atrial ZnT-1 expression and atrial fibrillation [10]. Other studies reported that down regulation of ZnT-3 and ZnT-10 can result in early senescence and cardiovascular diseases [11]. The syndromes of hepatic cirrhosis, dystonia, polycythemia, Parkinsonism, chronic liver disease and hypermanganesemia were found to be caused by mutations in ZnT-10 [12], [13]. In addition, increased risk of developing Type-II diabetes is associated with single amino acid polymorphisms of the human ZnT-8 [14]. The common allele of ZnT-8 contains Arg at position 325 and is considered to be the risk allele for acquiring type-II diabetes, whilst the less common allele, which contains Trp at the same position, provides some resistance. The R325 variant exhibited a lower zinc ion transport activity than the less abundant W325 variant [15]. The spatial localization of residue 325 is presumed to be in the cytosolic C-terminal domain (CTD) of ZnT-8, based on homology modeling [15].

In general, CDF transporters fold into two separate domains: a transmembrane domain (TMD) and a cytosolic domain [3], as presented by the determined structures of FieF (YiiP) from *Escherichia coli* [16] and a further, bacterial, FieF homolog from *Shewanella oneidensis* [17]. These structures were determined in a Zn^+2^-bound state and presented a homodimeric fold. Cation transport through the TMD is facilitated by conformational changes from the cytoplasm-facing conformation to a periplasm-facing conformation [17]. Other CDF CTDs structures were determined in either apo-form or Zn^+2^-bound forms and were found as stable, V-shaped dimers. These CTD structures include MamM from *Magnetospirillum gryphiswaldense*, CzrB from *Thermus thermophilus* and TM0876 from *Thermotoga maritime* [18]–[20]. The apo-form V-shaped dimer exhibits natural flexibility in solution that is attributed to a subtle equilibrium between two opposite local domain forces. These include charge repulsion between the two monomers and attraction via a hydrophobic interaction at the center of the internal dimerization interface, pulling the monomers closer [20]. MamM and CzrB CTD dimers exhibited a conformational change toward a tighter V-shaped dimer upon zinc binding that is considered to be associated with transport activation and regulation [19], [20]. The dimerization interface was shown to be highly significant to the CDF transport activity as the introduction of a mutation into the dimerization interface of MamM did not alter dimer formation but rather abolished the *in vivo* transport activity [20].

The CDF transporter MamM is a highly conserved protein within magnetotactic bacteria (MTB) [21]–[23]. MTB are an extraordinary group of prokaryotes that utilize CDF proteins for iron transport and biomineralization of magnetic iron nanoparticles within intracellular membrane-enclosed organelles called magnetosomes [24], [25]. In the genetically tractable alphaproteobacterium *M. gryphiswaldense*, the inorganic cores of magnetosomes consist of nanocrystals of magnetite (Fe_3_O_4_) that are aligned as intracellular chains to serve as a geomagnetic field sensor for navigation. MamM was recently proposed to function as a magnetosome-directed iron transporter required for iron biomineralization, since its deletion abolished magnetite biomineralization. Amino acid substitutions within the conserved TMD and CTD metal-binding sites resulted in alterations to magnetite crystal size, morphology and mineral type [20], [23]. Furthermore, these *in vivo* studies demonstrated the ability of MamM to form homo-dimers, as well as to stabilize MamB, an additional CDF homologue associated with the magnetosome membrane [23].

In this study we tested the ability of the magnetite biomineralizing bacterium *M. gryphiswaldense* to serve as an effective model system to study human CDF-related diseases. As a case study, we introduced ZnT-8 Type-II diabetes-associated mutations into the magnetosomal CDF protein MamM. By following the easily measurable phenotypic effects on the bacteria's magnetic response caused by altered iron deposition and magnetite biosynthesis, we provide here new structural and functional insights that are associated with the increased risk of developing Type-II diabetes.

## Results

**Figure 1:**
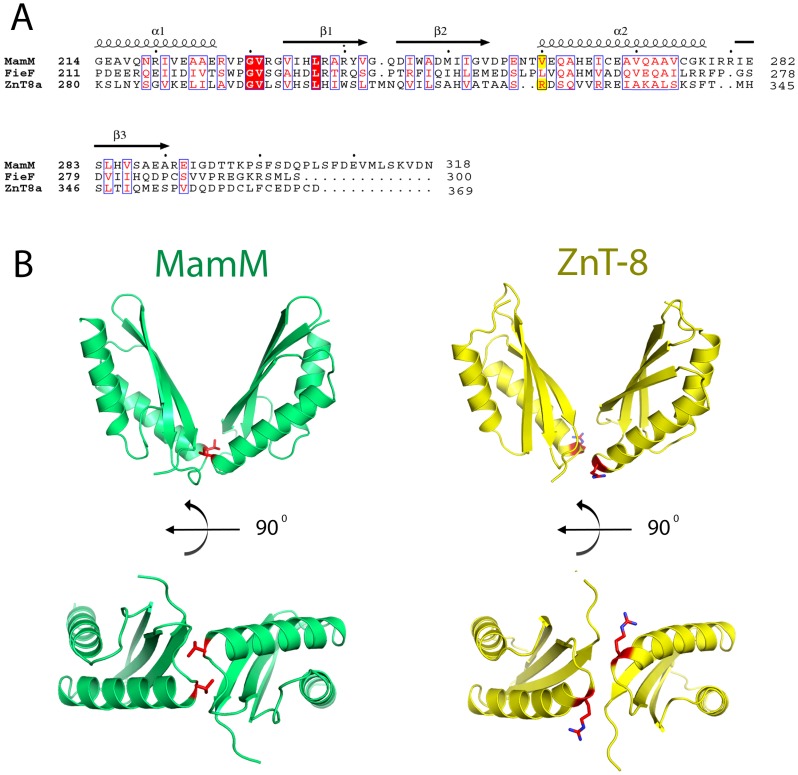
ZnT-8 homology model construction. (A) Multiple sequences and structural alignments reveal that the variable ZnT-8 position 325 overlaps Valine 260 of MamM protein. Valine 260 is located at the bottom of the V-shaped dimer and serves as the stand-alone hydrophobic interaction stabilizing the CTD dimerization interface. (B) ZnT-8-CTD homology model shares great structural similarity to MamM-CTD. Valine 260 of MamM and the representative arginine allele at position 325 of ZnT-8 are presented as red sticks.

In order to determine the positions of the Type-II diabetes-related ZnT-8 allele polymorphism within its bacterial homolog, MamM, we generated several homology models. These homology models were constructed using the HHPred server (http://toolkit.tuebingen.mpg.de/hhpred) and were based on multiple sequence alignment, structural alignment and previously determined CTD structures [16], [18], [19], [26], [27]. The resulting models shared great similarity with the MamM-CTD structure and the variable ZnT-8 position 325 overlaps MamM Val260 (Fig. 1A). In MamM, Val260 is located at the bottom of a V-shaped dimer on a loop connecting β-sheet 2 to α-helix 2 (Fig. 1B). The 260 position was shown to have a significant role in dimer stabilization, as the symmetric interaction between two Val260 belonging to two opposite monomers is the only hydrophobic interaction at the dimerization interface (Fig. 1B) [20].

### Human-related CDF mutations alter the bacterial magnetic response

**Figure 2:**
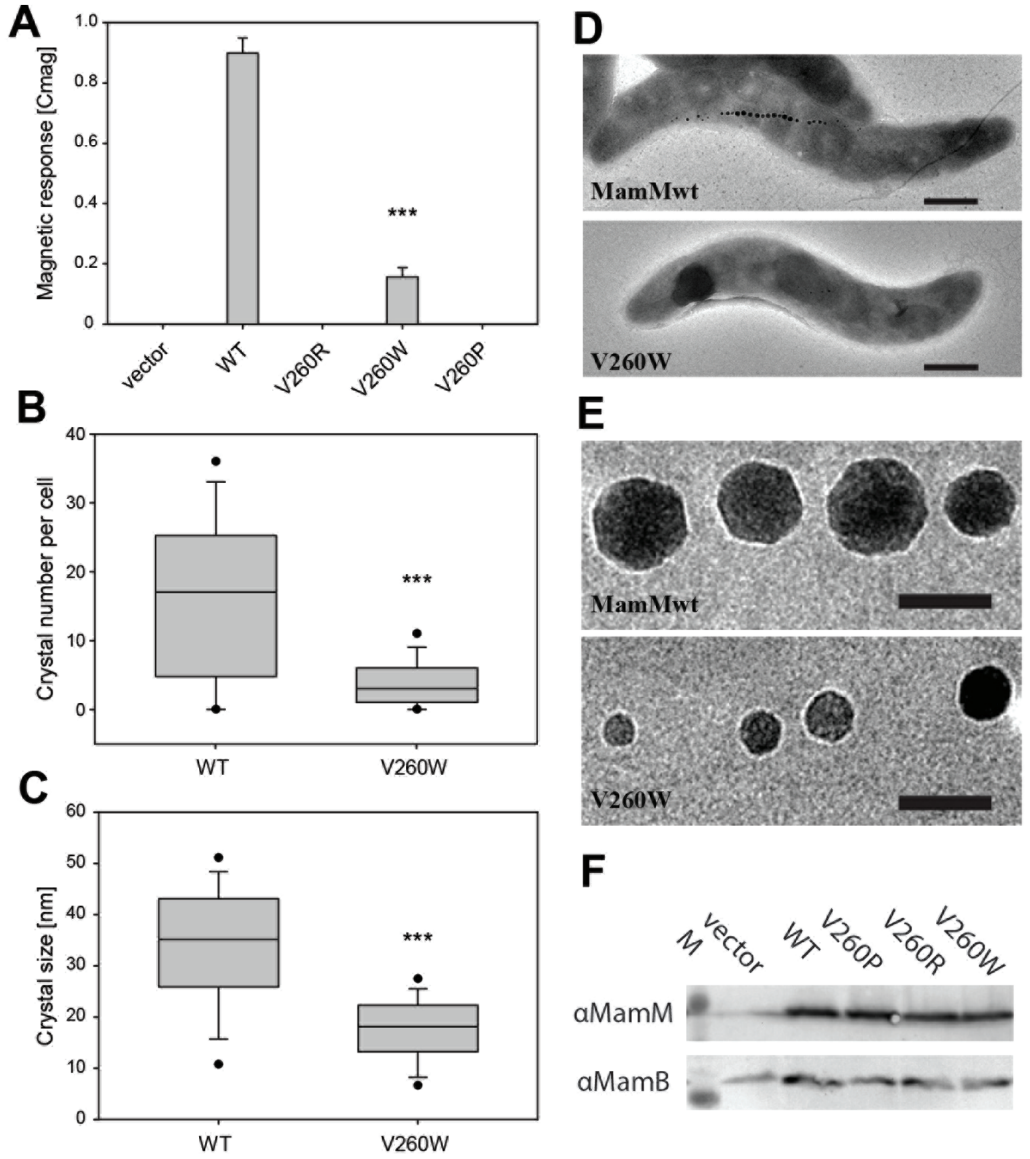
Effects of amino acid substitutions at the putative MamM-CTD dimerization interface on magnetic response, crystal number per cell, crystal size and crystal shape. (A) Magnetic response of Δ*mamM* strains expressing wild-type *mamM*, *mamM V260R, mamM V260W* and *mamM V260P*. Values are given as means ± standard deviations from ≥ 6 independent cultures. Statistical significance of alterations from the strain expressing wild-type *mamM* was tested using the *t*-test (***, *P*< 0.001). (B) Box plot showing the distribution of crystal numbers per cell from Δ*mamM* strains expressing wild-type *mamM* and *mamM V260W*. Statistical significance of alterations from the strain expressing wild-type *mamM* was tested using the Mann-Whitney test (***, *P*< 0.001). (C) Box plot showing the magnetite crystal size distribution of Δ*mamM* strains expressing wild-type *mamM* and *mamM V260W*. Statistical significance of alterations from the strain expressing wild-type *mamM* was tested using the Mann-Whitney test (***, *P*< 0.001). (D) Representative TEM images of cells expressing wild-type *mamM* and *mamM V260W*. Scale bar, 500 nm. (E) Representative TEM images of magnetite crystals from Δ*mamM* strains expressing wild-type *mamM* and *mamM V260W*. Scale bar, 50 nm. (F) Immunodetection of MamM and MamB wild-type and mutant proteins in cell lysates of trans-complemented Δ*mamM* strains. M, Molecular weight marker. MamB stability is not affected by MamM isoforms.

To study the functional effects of ZnT-8 CTD natural polymorphism on the transporter's activity, we generated MamM-CTD point mutations at position 260, similar to those at position 325 of ZnT-8 (Arg or Trp). Additionally, we tested Glycine and Aspartic acid mutations at position 260. First, we tested the effects of these point mutations on magnetite biomineralization *in vivo*. In previous *in vivo* studies, deletion of the *mamM* gene entirely abolished magnetosome biomineralization, whereas transcomplementation of the MSR-1 Δ*mamM* strain with *mamM* carrying substitutions of various single amino acid residues resulted in smaller and fewer crystals, as indicated by the gradual decrease in cellular magnetic response (C_mag_) [20], [23], [28]. In a similar manner, upon targeted mutagenesis of MamM, we monitored the resulting effects on magnetosome biomineralization as a sensitive proxy for iron uptake by following changes of the C_mag_, as well as by performing TEM analysis of the number, size and morphology of the formed electron-dense iron nanoparticles. Plasmid-derived transcomplementation of MSR-1 *ΔmamM* with the wild-type allele restored magnetosome formation to wild-type-like particle diameters (34 ± 12 nm) but with a reduced particle number per cell by ∼50% (16 ± 12 nm). As observed previously [23], the decrease in number of particles per cell was at least partially a consequence of a substantial number of cells within the population that remained non-magnetic (10-20%). Transcomplementation of MSR-1 *ΔmamM* cells with mutated MamM demonstrated that a magnetic response could be restored for V260G and V260W but not for the V260R and V260D mutants, which remained non-magnetic (Fig. 2A). Whereas MamM V260G restored the magnetic response to almost wildtype-levels (C_mag_  =  0,86 ± 0.03), MSR-1 *ΔmamM* cells harboring the MamM V260W plasmid displayed significantly reduced magnetic responses (C_mag_  =  0.16 ± 0.03; P< 0.001, *t*-test), lower crystal numbers per cell (3.8 ± 3.6; P< 0.001, Mann-Whitney test) as well as a reduced average crystal size (17.7 ± 6.2 nm; P< 0.001, Mann-Whitney test), indicating a reduced MamM transport activity (Fig. 2B-E; Table B in File S1). Expression levels of both Type-II diabetes mutants were found to be similar to wild-type MamM, as were their abilities to stabilize MamB protein expression (Fig. 2F).

### The mutated C-terminal domains are stable dimers that bind zinc

**Figure 3:**
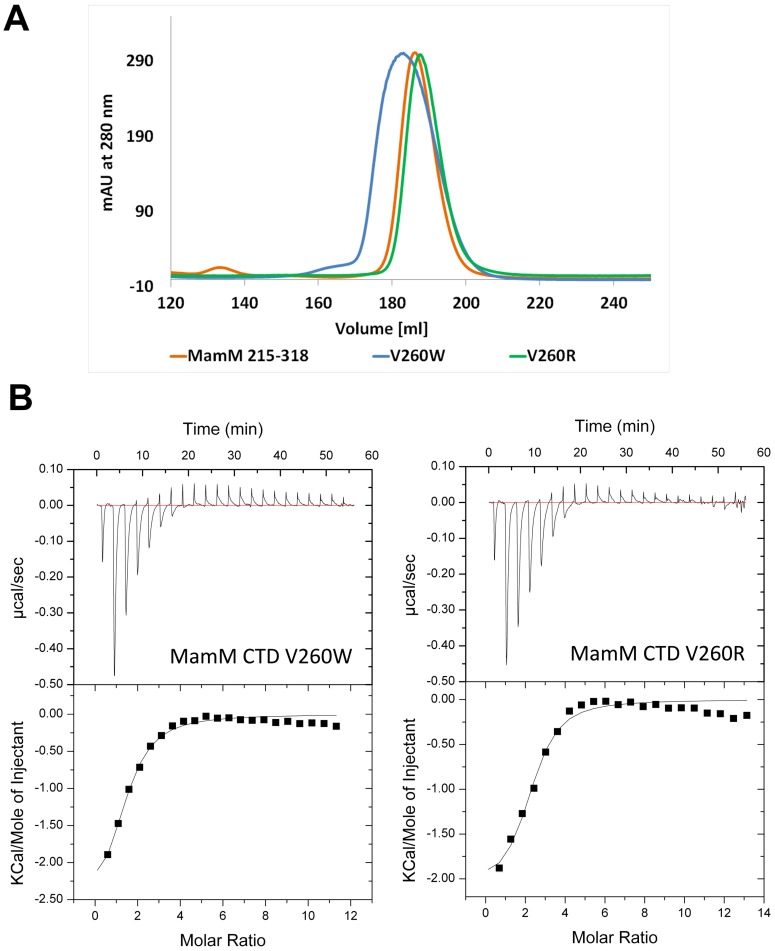
MamM-CTD and Type-II diabetes-related mutants are dimers that bind zinc. (A) MamM-CTD and Type-II diabetes-related mutants elute form size-exclusion chromatography (Superdex200) in a volume appropriate for dimers. (B) Zn^2+^ pH-dependent binding of the MamM-CTD mutants. ZnCl_2_ (5 mM) was titrated into MamM-CTD V260W (92 µM) and MamM-CTD V260R (91 µM) in 1.8 µl aliquots every 150 s. Measurements were performed at 25°C in 10 mM Tris·HCl pH 8.0, 150 mM NaCl. Top panels show the heat change during injection and bottom panels represent the data after peak integration. Data were fit using the Origin software. See Table 1 for the binding parameters.

To confirm that these observed *in vivo* phenotypes represent true functional effects we characterized the recombinant MamM-CTD V260R and V260W mutants by several *in vitro* methodologies [20]. Considering the similar binding affinities of MamM-CTD for both iron and zinc cations [20], when needed during our experiments, we used the stable zinc ions instead of ferric ions, as the latter form insoluble iron hydroxides at alkaline pH. Firstly, size exclusion chromatography (SEC) clearly demonstrates that both mutants maintain the dimerization ability, as both elution volumes are similar to wild-type MamM-CTD (Fig. 3A). Notably, the SEC elution profile of the V260W mutant presents a slightly wider peak but does not reach the size of a trimer. This extended elution range can be attributed to the introduction of the large side chain of Trp to the tight dimerization interface, which eventually leads to an increase in the dimer globular diameter. Secondly, isothermal titration calorimetry (ITC) experiments reveal that both mutants can still bind zinc cations (Table 1). ITC results indicated that the number of zinc binding sites was reduced for the V260W mutant (2.7 ± 0.2) compared to the V260R mutant (4.4 ± 0.3) and wild-type MamM-CTD (4.1 ± 0.2) (Fig. 3B).

**Table 1:** Thermodynamics of zinc binding to MamM-CTD dimer and mutants, as measured by Isothermal Titration Calorimetry.

Protein	N	K_d_	ΔH	ΔS	ΔG
		(µM)	(kcal/mol)	(cal/mol/deg)	(kcal/mol)
MamM-CTD	4.1 ± 0.2	16 ± 4	-1.8 ± 0.1	16	-4.77
MamM-CTD V260W	2.7 ± 0.2	37 ± 9	-2.8 ± 0.3	10.9	-3.25
MamM-CTD V260R	4.4 ± 0.3	19 ± 7	-2.1 ± 0.2	14.4	-4.30

### The crystal structure of the risk-associated mutant allele presents a twisted dimer

**Figure 4:**
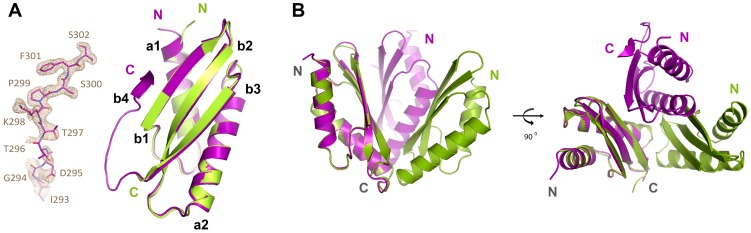
Structural overlay of the wild type MamM-CTD in green and V260R mutant in purple. (A) Monomers overlay present a similar metallochaperone-like fold. In contrast to wild type MamM-CTD structure that presents a flexible C-terminal tail, one of V260R mutant monomers presents an additional beta-sheet (b4). A 2.05 Å 2F_o_ – F_c_ electron density omit map was calculated and is presented around b4. The map is countered at 1.0 σ (light brown). (B) Dimer packing overlay reveals altered and twisted dimer packing for V260R mutant.

**Figure 5:**
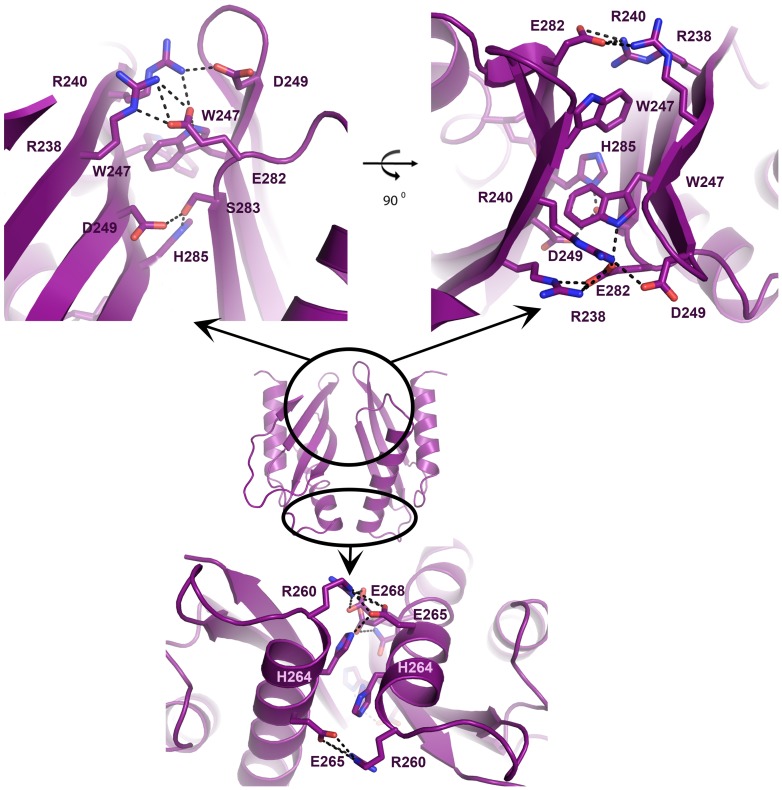
Altered dimerization interface of V260R mutant. *(Top)* The interactions at the top of the V-shaped dimer are maintained by a hydrophobic core of the two symmetrical Trp247 surrounded by two polar interaction patches. The first polar patch includes Arg238-Glu282 salt bridge with additional polar interactions to Asp245 side chain and the backbone carbonyl of Val242. The second patch includes a double Arg238-Glu282-Arg240 salt bridge. *(Bottom)* The interactions at the bottom of the V-shaped dimer include two interaction patches, the first is stabilized by an Arg260-Glu265 salt bridge with additional polar interactions to the side chains of Glu268 and His264. The second, lower, interaction patch is stabilized by a single Arg260-Glu268 salt bridge.

Crystallization trials for both mutants resulted only in a single crystal form of the V260R mutant. The X-ray determined structure of MamM-CTD V260R contained two tightly packed monomers in the asymmetric unit (Table A in File S1). Each monomer adopts the typical CDF-CTD metallochaperone-like fold which contains two α-helices and three β-sheets (Fig. 4A). Overlapping the monomers of wild-type MamM-CTD and the V260R mutant demonstrated that both share a similar fold, with a Cα-RMSD of 0.43 Å (Fig. 4A). While the wild-type MamM-CTD structure contains a disordered C-terminal tail (residues 293-318), a rigid conformation was adopted by residues 294-302 in a single V260R monomer comprising the dimer. This rigid C-terminal extension gave rise to the formation of a fourth β-sheet aligned in parallel to the three existing β-sheets located in the inner metal-binding interface of the dimer (Fig. 4A). The most significant observation derived from the V260R mutant crystal structure was the altered dimeric packing. The V260R dimer presented a tighter and twisted conformation in reference to wild-type MamM-CTD dimeric structure (Fig. 4B). These dimeric fold alternations could have been driven by crystal packing or as the result of the V260R mutation. As such, the V260R dimer structure presents a dramatic increase in the dimerization interface surface (377 Å^2^), in reference to the dimerization interface surface of the wild-type (193 Å^2^). According to the V260R determined structure, the altered dimerization interface is stabilized asymmetrically by several salt bridges, polar and hydrophobic interactions located at the top and bottom of the dimer (Fig. 5). The interactions at the bottom of the V-shaped dimer include two interaction patches; an Arg260-Glu265 salt bridge stabilizes the first, with additional polar interactions to the side chains of Glu268 and His264. The second, bottom, interaction patch is stabilized by a single Arg260-Glu268 salt bridge. The interactions at the top of the V-shaped dimer are maintained by a hydrophobic core of the two symmetrical Trp247's surrounded by two polar interaction patches. The first, top, polar interaction patch includes the Arg238-Glu282 salt bridge with additional polar interactions with the Asp245 side chain and the backbone carbonyl of Val242. The second, top, polar interaction patch includes a double Arg238-Glu282-Arg240 salt bridge (Fig. 5).

**Figure 6:**
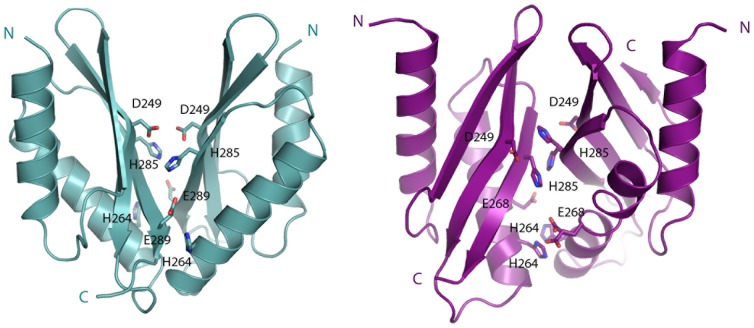
Metal binding sites of MamM-CTD and V260R mutant. MamM-CTD presents a central binding site formed by symmetrical Asp249-His285 and two symmetrical peripheral binding sites formed by His264-Glu289. The twisted dimeric fold of V260R allows the formation of the central putative binding site and two alternative peripheral symmetrical binding sites between Glu268-His264, each from a different monomer.

Three putative metal binding sites were previously described for MamM-CTD: a central binding site formed by symmetrical Asp249-His285 and two symmetrical peripheral-binding sites formed by His264-Glu289 (Fig. 6) [20]. The twisted dimeric fold of V260R allows the formation of the central putative binding site but prevents the formation of the wild-type His264-Glu289 double peripheral binding sites. However, by examining the V260R dimer, we could predict two alternative peripheral symmetrical binding sites between Glu268-His264, each from a different monomer (Fig. 6). In addition to the central binding site that accommodates two zinc cations, these two alternative peripheral binding sites of the twisted V260R dimer might explain the ability of the V260R dimer to bind four zinc cations *in vitro*.

### *In silico* fold stability of MamM mutants

**Figure 7:**
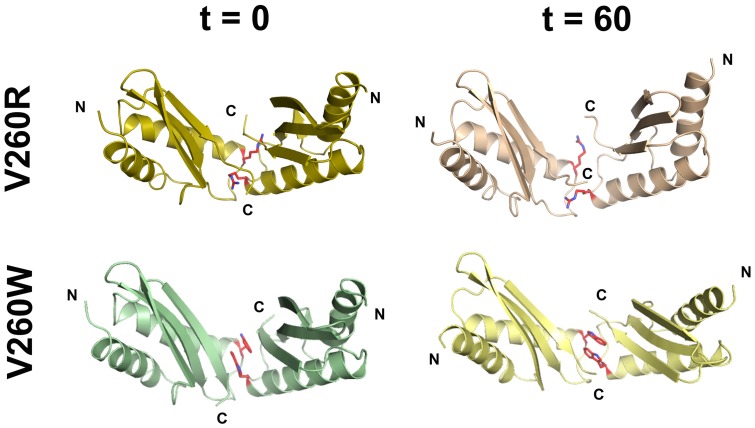
The structural V260R/W models show a stable dimerization interface and a V-shaped fold along MD simulations. Initial models (t  =  0) were constructed according to the wild type MamM. Final simulated structures (t  =  60 ns) show a stable V-shaped fold. Mutated residues are depicted as red sticks.

To test whether the V260R dimer fold alternations could be driven by tight crystal packing and contacts, we examined the dimer generated in the crystal asymmetric unit by Molecular Dynamics (MD) simulations to see if it can persist as a stable dimer in solution. The twisted dimeric fold of V260R was shown to be stable along the 60 nsec of the simulations (Fig. A in File S1). Next, we evaluated the typical V-shaped CTD dimer's ability to maintain a stable dimeric fold upon introduction of Arg or Trp mutations. Two mutated V260R and V260W structural models were designed and minimized according to the V-shaped dimer fold presented in the apo-MamM-CTD determined structures (PDB codes: 3W5Y, 3W5X). The two structural models had been used as initial structures for MD simulations (Fig. 7). Additionally, these MD simulations were provided with a maximum dimer N-termini distance restrain of 60 Å that was set to mimic the dimeric-related restrains provided by the TMD. Analysis of these simulations included distance measurement between the Cα atoms of two pairs of identical residues from each monomer and Cα atoms of four residues to monitor the dihedral angle. The first pair was Arg240-Arg240 located at the top of the V-shaped dimer, whilst the second pair was Pro256-Pro256 located at the dimer interface at the bottom of the V-shape [20]. The MD simulations analyses demonstrated a stable dimer fold for both Arg and Trp mutants throughout the 60 nsec simulations, similar to wild-type MamM-CTD (Fig. A in File S1). In addition, both V260W and V260R mutants presented an increased dihedral angle and Arg240-Arg240 distance in reference to the wild type MamM-CTD (Fig. A in File S1). These observed fold changes are probably the result of the introduction of the large side chains of Arg and Trp into the tight dimerization interface formed by the original Val260 residues (Fig. 7). Remarkably, the MD simulation of the V260R mutant model did not adopt the observed tight dimer packing seen in the crystal structure. This result is also supported by *in vitro* Small Angle X-ray scattering (SAXS) measurements of V260R, V260W and V260P mutants which exhibit similar wild-type-like curves (data not shown).

**Figure 8:**
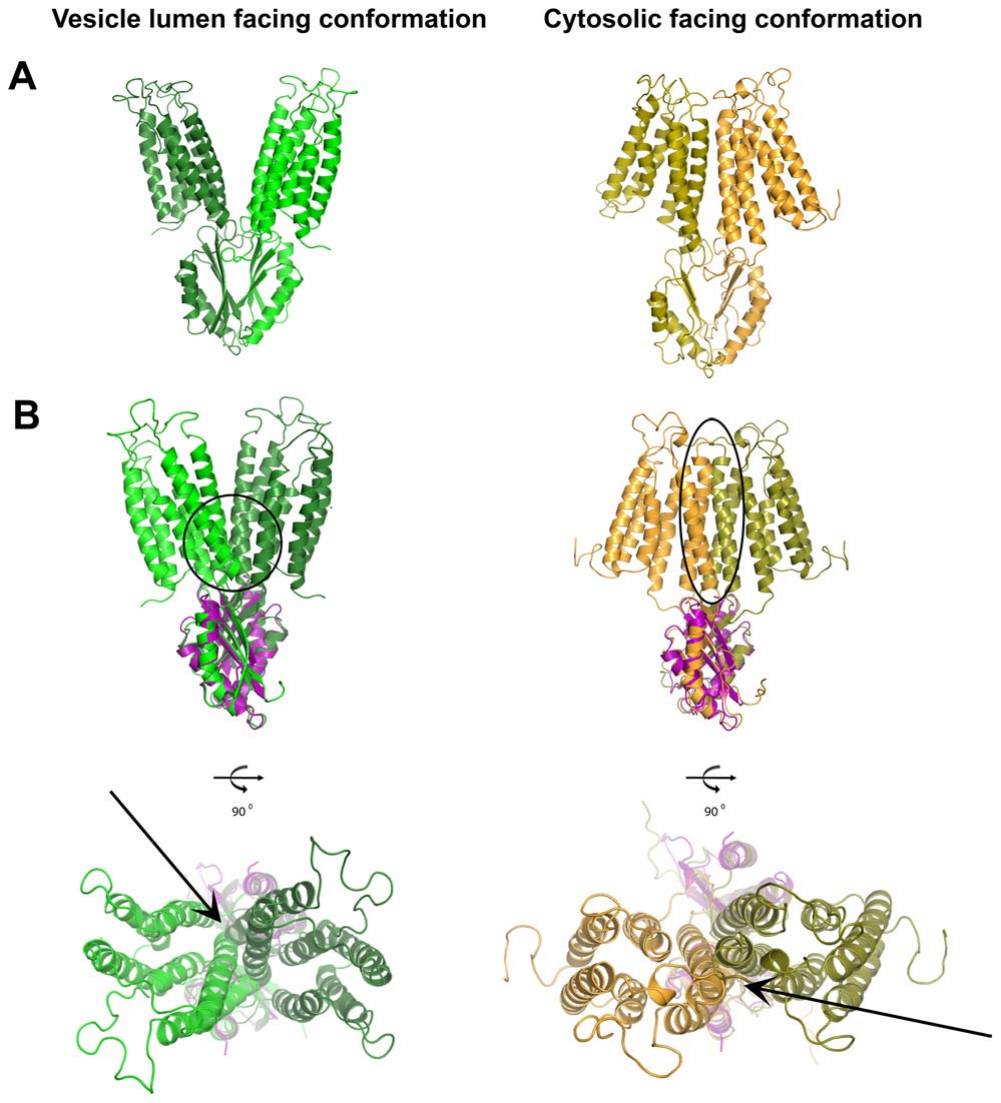
The effect of a twisted CTD fold on the TMD. Superposition of full-length monomers of FieF (PDB code: 3H90 – monomers are in two shades of green) and YiiP homolog (PDB code: 3J1Z – monomers in two shades of orange) according to the twisted CTD packing presented by V260R, in purple. These clashes are present in both (A) the TMD vesicle lumen-facing transport conformation and (B) the cytosolic-facing transport conformation. Backbone clashes are highlighted by circles and arrows.

To discriminate between the two possible folds of the V260R CTD dimer we examined the effects of these different CTD folds on the TMD. Superposition of the full length FieF (PDB code: 3H90) and YiiP homolog (PDB code: 3J1Z) monomers, according to the twisted CTD packing presented by V260R, revealed severe clashes within the TMD. These clashes are present in both the TMD vesicle lumen-facing transport conformation and cytosolic-facing transport conformation (Fig. 8). Considering the nature of these TMD clashes, we propose that if such a twisted dimer fold exists *in vivo*, it might alter the TMD ion transport ability.

## Discussion

In this study we used the magnetotactic bacterium *M. gryphiswaldense* as a simple, tractable system to test structural and functional effects of mutations within CDF homologs that were identified to be disease-associated in humans, specifically Type II diabetes. Upon introduction of homologous mutations into the magnetosome-associated CDF transporter MamM, the *in vivo* effects can easily be characterized by measurements of magnetite crystal size and morphology, as well as by the rate of magnetic response acquisition of iron-starved cells [20]. These effects can later be translated into mechanistic knowledge of the phenotypic alternations caused by the relevant mutations. The human ZnT-8 protein was chosen to serve as a case study as it presents a single amino acid polymorphism at residue 325 that is located at the CTD dimerization interface. A previous study demonstrated that the ZnT-8 R325 variant exhibited a lower zinc ion transport activity than the less abundant W325 variant [15]. According to our *in vivo* results the W325 allele of ZnT-8 can restore some of the magnetic response of the MTB as it allows the spring-like associated motion of the CTD [20] by forming π-interactions between opposing Trp side chains from the two monomers. On the contrary, the R325 allele or MamM V260D provide a less hydrophobic dimerization interface and might accommodate multiple side chain conformations. Moreover, the arginine side chain is highly susceptible to salt bridge formation with the abundant negatively charged residues found in cation binding proteins (for example, the MamM-CTD contains 18% Asp and Glu residues), while Asp side chain at position 260 can result in negative charge repulsion. The formation of such undesirable salt bridges and possible charge repulsion can lead to severe alternations in the dimerization interface. As a result, the suggested induced conformational shift upon cation binding which provides the activation signal from the CTD to the TMD might be inhibited and proper CTD motion will be prevented. One example of such an altered conformation was seen in the V260R determined structure. Although the V260R mutant dimer was able to bind the same number of zinc cations as the wild-type dimer *in vitro*, it was not functional *in vivo*. Hence, for the ZnT-8 R325 variant the proper activity of the sensory CTD domain is not allowed and later prevents proper transport by the TMD of ZnT-8. Therefore, the ZnT-8 case study results suggest that other ZnT mutation's effects can be examined using the magnetosome biomineralization system.

In our previous study, position 260 of MamM was identified as a vital element for proper cation transport [20]. The introduction of the V260P mutation led to a non-magnetic *in vivo* phenotype similar to the described V260R and V260D mutants. Additional *in silico* comparisons demonstrate that the V260W mutant exhibits a similar distance increase at the top of the V-shaped dimer and an increase of dihedral angle as presented by V260P mutant. Biophysical measurements present an increase in the number of zinc cations bound for both V260R and V260W mutants, compared to V260P. Although V260R and V260W both presented some similarities to the V260P mutant in several measures, none of them presented an overall identical effect. For example, the *in vitro* measurements demonstrate that V260W is more similar to V260P than V260R. However, the V260W mutant can restore magnetic behavior whilst V260R and V260P cannot. Considering these new Type-II diabetes related mutants as well as V260G and V260D mutants, our results further support our previous suggested CDF activation model, in which cation binding to the CTD induces conformational changes towards a tighter and more compact fold, which allows for the activation of iron transport through the TMD [20]. The CTD movement was described as a spring motion driven by the negatively charged V-shaped "arms" which are drawn apart due to charge repulsion while the hydrophobic interactions at the dimerization interface push the "arms" closer toward a tolerable and favorable distance which maintains stable hydrophobic interactions. Hence, the CTD functionality depends on the subtle equilibrium between charge repulsion and hydrophobic interactions. Therefore, we believe that although having a large side chain, Trp can maintain the domain function as it can adopt favorable π-stacking stable interactions and thus allows the CTD spring-like motion.

The crystal structure of V260R provides an interesting structural observation for the flexible C-terminal tail that bears possible implications for CDF protein-protein interactions. Eight out of 25 residues comprising the flexible C-terminal tail were found to adopt a discrete beta and coil fold in the V260R mutated structure. The spatial alignment of this fourth, additional, beta-sheet with the three other beta-sheets comprising the metallochaperone-like CTD fold may promote interaction with other proteins, such as, for instance, cellular metallochaperones or other CDFs. An example for such a possible mode of interaction can be a beta-sheet bridge that interconnects the CTDs of the two magnetosome CDF proteins, MamM and MamB, and thus stabilizes the MamB protein [23]. This interaction is further supported by a similar tail structure in a different MamM D249A&H285A double mutant (PDB code: 3W8P) [20]. Overall, we demonstrated that the combination of multi-disciplinary approaches with a unique bacterial biomineralization system provides a promising alternative system to study the mechanism of CDF-related human diseases.

## Methods

### Protein expression, purification and site-directed mutagenesis

Performed as previously described [20], [29], [30].

### Structure determination

34 mg/ml of purified MamM-CTD V260R mutant was crystallized using the vapor diffusion methodology at 20°C (0.1M Tris pH = 7.0, 15% PEG 3350, 0.2M MgCl). The crystal was harvested after the addition of 50% polyethylene glycol 3350 cryo-protecting solution and flash-cooled in liquid nitrogen. Data collection was performed for a single crystal in 100 K at ID14-4 beamline, European Synchrotron Radiation Facility (ESRF), Grenoble, France. Data were reduced and scaled using the HKL2000 suite [31]. Phases were obtained using Phaser molecular replacement and PDB code: 3W6X as a template [19]. The final model was built by Coot [32] and refined in REFMAC [33]. For Rfree calculation, 5% of the data were excluded. Structural figures were prepared with PyMOL [34].

### Least-squares overlaps

R.M.S. calculations were performed with SwissPDB viewer [35] using the domain alternate fit to align structures on the basis of the conserved domain and to define the conformational changes of the structural homologues.

### Isothermal titration calorimetry

Isothermal titration calorimetry measurements were performed on an iTC200 calorimeter (Microcal, GE Healthcare) at 25°C. Both protein and zinc chloride were diluted to the same final buffer of 10 mM Tris·HCl, pH 8.0, 150 mM NaCl. Aliquots (1.8 µl) of the zinc chloride solution (5 mM) were titrated every 150 sec. The data were fit using ORIGIN 7.0 software (Origin Lab) to the single-site binding isotherm. The integrated peak of the first injection was excluded from the fit due to the large errors in the first step.

### Bacterial strains, oligonucleotides and plasmids for *in vivo* characterization

Bacterial strains, oligonucleotides and plasmids used in this study are listed in Table C in File S1. All strains were cultivated as described previously [20], [36].

### Trans-complementation of *ΔmamM*

For trans-complementation assays, pRU1 and *mamM* containing derivatives were transferred to *ΔmamM* by conjugation. After plasmid transfer the average magnetic response (C_mag_) of three independent trans-conjugants was assayed [28]. Therefore, cells were aligned at different angles relative to a light beam by means of an external magnetic field. The ratio of the resulting maximum and minimum scattering intensities (*C*_mag_) is correlated with the average number of magnetic particles. Imaging of trans-complemented cells by transmission electron microscopy (TEM) was performed as previously described [37]. Briefly, unstained cells were adsorbed on carbon coated copper grids, air-dried (Plano, Wetzlar), and analyzed with a FEI Tecnai F20 transmission electron microscope (FEI; Eindhoven, the Netherlands) at an accelerating voltage of 200 kV. Images were captured with a FEI Eagle 4096 × 4096 pixel CCD camera using EMMenue 4.0 and FEI's Explore 3D. Expression of *mamM* and site-directed variants was confirmed by separation of 10 µg of whole cell protein by SDS-polyacrylamide (12%) gel electrophoresis (PAGE) and subsequent Western blot analysis, as previously described [23].

### Classical Molecular Dynamics (MD) simulations

#### Experiment-based MamM protein models' construction

For the classical MD simulations, we applied three models in this study. We used the crystallography structures of the wild-type MamM-CTD for the molecular dynamics (MD) simulations for the open state. We performed V260R and V260W mutations to form two structural mutated models. We further used the crystallographic structure of the V260R mutant. The three models were first minimized, as performed previously for Aβ and tau oligomers [38]–[41].

#### Molecular dynamics (MD) simulations protocol

MD simulations of the solvated variant mutant models of the protein were performed in NPT (N, number of particles; P, pressure and T, temperature) ensembles using the NAMD program [42] with the CHARMM27 force-field [43], [44] for 60 ns. The models were explicitly solvated with TIP3P water molecules [45], [46]. The Langevin piston method [42], [47], [48] with a decay period of 100 fs and a damping time of 50 fs was used to maintain a constant pressure of 1 atm. The temperature (310 K or 318 K) was controlled by a Langevin thermostat with a damping coefficient of 10 ps^-1^ [42]. The short-range Van der Waals (VDW) interactions were calculated using the switching function, with a twin range cutoff of 10.0 and 12.0 Å. Long-range electrostatic interactions were calculated using the particle mesh Ewald method with a cut-off of 12.0 Å for all simulations [49],[50]. The equations of motion were integrated using the leapfrog integrator with a step of 2 fs. All initial variant models were energy minimized and then solvated in a TIP3P water box with a minimum distance of 15 Å from any edge of the box to any protein atom. Any water molecule within 2.5 Å of the protein was removed. Counter ions (Na^+^ or Cl^-^) were added at random locations to neutralize the protein's charge. The solvated systems were energy minimized for 2000 conjugated gradient steps. The counter ions and water molecules were allowed to move. The minimized solvated systems were heated at 200 K, where all atoms were allowed to move. The systems were then heated from 200 K to 250 K for 300 ps and equilibrated at 310 K or 318 K for 300 ps. All simulations ran for 60 ns and structures were saved every 10 ps for analysis. These conditions (310 K or 318 K and 60 nsec of timescales) were applied to test the stabilities of all the variant models.

### Coordinates

The structure has been submitted to the Protein Data Bank (3W8G).

## Supporting Information

File S1 Supporting information figures and tables.
(PDF)
