# Supplementary material for: Bacterial Magnetosome Biomineralization - A Novel Platform to Study Molecular Mechanisms of Human CDF-Related Type-II Diabetes
Source: PLoS One. 2014 May 12;9(5):e97154. doi: 10.1371/journal.pone.0097154 (PMC4018254; doi:10.1371/journal.pone.0097154)
Supplement: File S1 — Supporting information figures and tables. (PDF) [file pone.0097154.s001.pdf]

Supporting Information for:

Bacterial Magnetosome Biomineralization - a Novel Platform to Study Molecular Mechanisms of Human CDF-related Type-II Diabetes

Natalie Zeytuni[*, †, ‡], René Uebe[¶¶, ‡], Michal Maes[**], Geula Davidov[*, †], Michal Baram[§, #], Oliver Raschdorf [¶¶], Assaf Friedler[**], Yifat Miller[§, #], Dirk Schüler[¶¶] and Raz Zarivach[*, †, ##].

[*]Department of Life Sciences and [†]The National Institute for Biotechnology in the Negev, Ben Gurion University of the Negev, Beer-Sheva, Israel.

[§]Department of Chemistry, Ben Gurion University of the Negev, Beer-Sheva, Israel.

[#]Ilse Katz Institute for Nanoscale Science and Technology, Ben-Gurion University of the Negev, Beér-Sheva, Israel

[**]Institute of Chemistry, The Hebrew University of Jerusalem, Givat Ram, Jerusalem, Israel.

[¶¶]Ludwig Maximillian University of Munich, Dept. Biology I, Martinsried, Germany.

[‡]These authors contributed equally to this work.

[##]Correspondence should be addressed to Raz Zarivach, Department of Life Sciences, Ben Gurion University of the Negev, Beer-Sheva, Israel. Tel: +972-8-6461999, Fax: +972-8-6472970, Email: zarivach@bgu.ac.il

Table A - Data collection and refinement statistics.

PDB code	3W8G
Protein	MamM CTD V260R
**Data collection**	ID14-4 - ESRF
Space group	$P6_1$
Cell dimensions	
a, *b*, *c* (Å)	68.48, 68.48, 56.40
α, β, γ (°)	90, 90, 120
Resolution (Å)	2.05
*R*sym or *R*merge	14.4 (51.3)
I / σ*I*	20.2 (4.75)
Completeness (%)	93.3 (99.8)
Redundancy	6.3 (5.0)
Wavelength (Å)	0.977
**Refinement**	
Resolution (Å)	2.05
No. reflections	8884
*R*work / *R*free	18.38/25.76
No. atoms	
Protein	627
Ligand/ion	88
Water	747
B-factors	
Protein	25.57
Ligand/ion	29.69
Water	33.38
R.m.s. deviations	
Bond lengths (Å)	0.0139
Bond angles (°)	1.7015

Values in parentheses are for the highest resolution shell.

Table B - Crystal number and size in trans-complemented Δ*mamM* cells

Parameter	Δ*mamM* (pRU1-mamMx)	
	WT	V260W
**Crystal number per cell**		
sample size	362	159
mean value	16.3	3.8
standard deviation	12.4	3.6
**Crystal size [nm]**		
sample size	1452	515
mean value	33.8	17.7
standard deviation	12.1	6.2

Table C – Bacterial strains, oligonucleotides and plasmids for in vivo characterization.

Strain/Oligo/Plasmid	Important features/Sequence	Source or reference
E. coli		
DH5α	F' Φ80d*lac* ΔM15 Δ(*lacZYA-argF*)U169 *deo*R *rec*A1 *end*A1	Invitogen
BW29427	*thrB1004 pro thi rpsL hsdS lacZ*ΔM15 RP4-1360 Δ(*araBAD*)567 Δ*dapA1341::[erm pir* (wt)]	Obtained from Datsenko, K. and Wanner B. L.
M. gryphiswaldense		
MSR-1 R3/S1	Rif^r, Sm^r spontaneous mutant, wildtype	(Schultheiss *et al*, 2005)
Δ*mamM*	R3/S1 but Δ*mamM*	(Uebe *et al*, 2011)
**Oligonucleotide**		
MamMV260W_for	AACACCTGGGAACAGGCGCAT	this study
MamMV260W_rev	CTCGGGATCGACGCCAATAA	this study
MamMV260R_for	AACACCCGCGAACAGGCGCAT	this study
**Plasmid**		
pRU1	pBBR1MCS-2 with P_{mamAB}	(Uebe *et al.*, 2011)
pRU1-mamMwt	pRU-1 + *mamM*	(Uebe *et al.*, 2011)
pRU1-mamMV260R	pRU-1 + *mamM V260R*	this study
pRU1-mamMV260W	pRU-1 + *mamM V260W*	this study

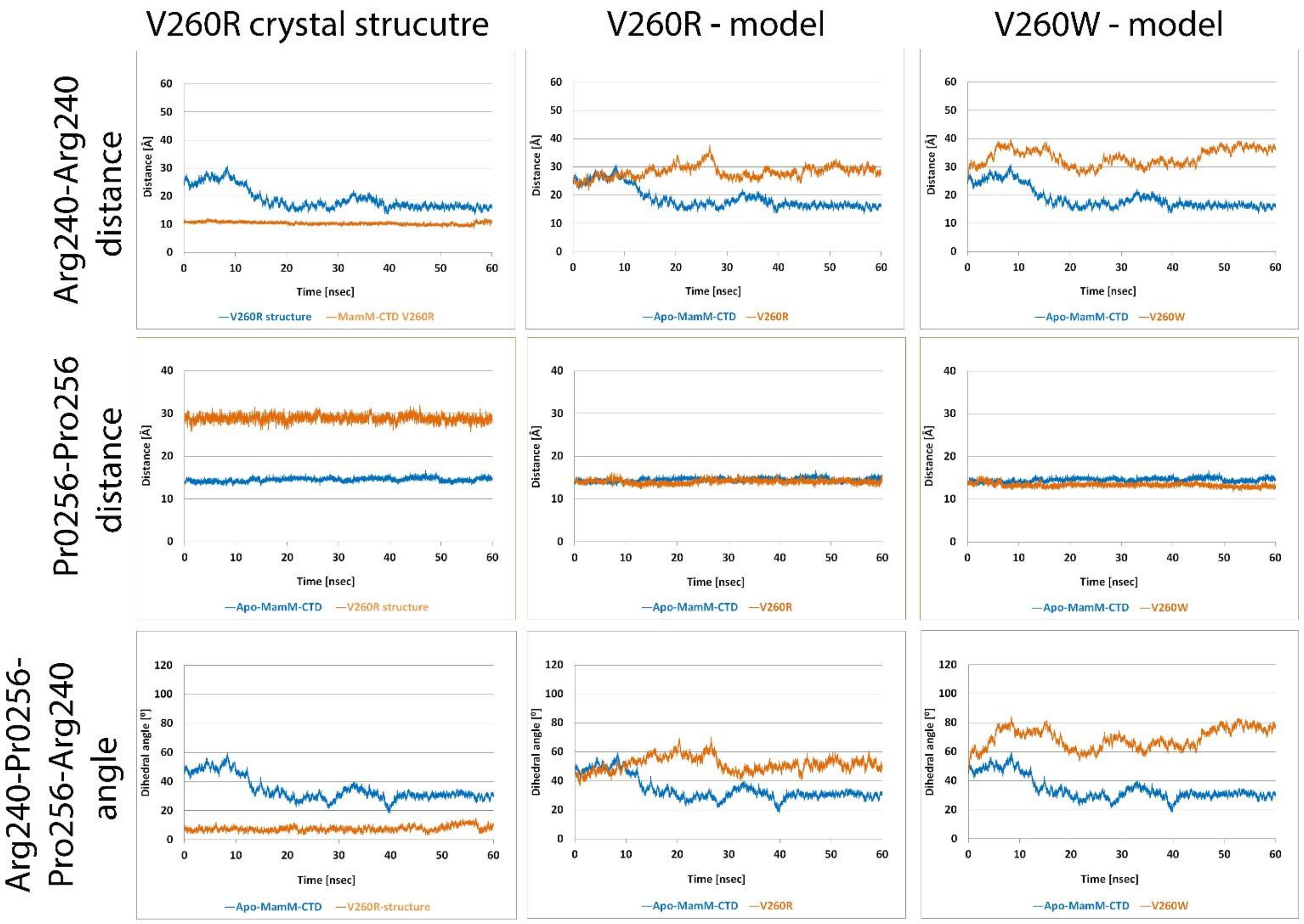

Fig. A – Molecular dynamics simulations analysis of different MamM-CTD mutants at position 260. The figure represents four different mutated MamM-CTD simulation inputs in 37ºC: V260R according to the packing observed at the determined crystal structure and three apo-MamM-CTD structures with modeled V260R/W mutations. The distances between the Cα of Arg240-Arg240 and Pro256-Pro256 as well as the dihedral angle of Cα Arg240-Pro256-Pro256-Arg240 were monitored throughout the 60 nsec simulations (orange) and compared to wild type apo-MamM-CTD simulation (blue). To mimic the effect of TMD on the CTD a 60 Å N-terminals dimer distance restrain was inserted to all simulations. The twisted and tight dimer conformation observed at V260R determined structure maintains a stable and tight dimer conformation throughout the simulation. The V260W/R mutated apo-MamM-CTD models present increased dihedral angle and Arg240-Arg240 distance in reference to the wild type MamM-CTD and maintain stable dimerization interface at the bottom of the V-like shape.
